# A randomized controlled trial of the dissemination of an mHealth intervention for improving health outcomes: the WiseApp for Spanish-speakers living with HIV study protocol

**DOI:** 10.1186/s12889-023-17538-y

**Published:** 2024-01-17

**Authors:** Felix Olaya, Maeve Brin, Pamela Baez Caraballo, Mina Halpern, Haomiao Jia, Sergio Ozoria Ramírez, Janeth Juarez Padilla, Samantha Stonbraker, Rebecca Schnall

**Affiliations:** 1 School of Nursing, Columbia University, 560 W 168th St, New York, NY 10032 USA; 2 Clínica de Familia La Romana, La Romana, Dominican Republic; 3 NYU Steinhardt School of Culture, Education, and Human Development, New York, NY 10003 USA; 4 NYU School of Medicine, New York, NY 10016 USA; 5 University of Colorado College of Nursing, Anschutz Medical Campus, Aurora, CO 80045 USA

**Keywords:** Medication adherence, People with HIV (PWH), Antiretroviral therapy (ART), Mobile Health (mHealth), Spanish speakers, Latinos

## Abstract

**Background:**

While there is no cure for HIV, adherence to antiretroviral therapy can extend the lifespan and improve the quality of life of people with HIV. Despite the global reduction of HIV infection rates in recent years, New York City and La Romana, Dominican Republic, continue to report high infection rates among Latino populations. Many people with HIV remain virally unsuppressed in these geographic hotspots, suggesting a need for additional interventions to overcome medication adherence barriers. Tailored and culturally appropriate mobile health (mHealth) technology can be an engaging way to improve adherence. The primary objective of this trial is to test the effectiveness of an mHealth tool to improve HIV medication adherence among Spanish-speaking people living in New York City and the Dominican Republic.

**Methods:**

The WiseApp study is a two-arm randomized controlled trial among 248 people with HIV across the New York and Dominican Republic sites over the course of 12 months. Participants are randomly assigned to either receive a CleverCap pill bottle that is linked to the WiseApp (intervention) or standard of care (control). All participants complete surveys at baseline, 3-month, 6-month, and 12-month follow-up visits and the study team obtains HIV-1 viral load and CD4 count results through blood draw at each study timepoint.

**Discussion:**

The use of mHealth technologies to improve medication adherence among people with HIV has been implemented in recent years. Although some studies have found improvement in adherence to antiretroviral therapy in the short term, there is limited information about how these interventions improve adherence among Spanish-speaking populations. Disproportionate rates of HIV infection among Latinos in New York City suggest an existing inequitable approach in reaching and treating this population. Due to a lack of mHealth studies with Latino populations, and apps tailored to Spanish-speakers, the WiseApp study will not only demonstrate the effectiveness of this particular mHealth app but will also contribute to the mHealth research community as a whole.

**Trial registration:**

This trial was registered with Clinicaltrials.gov (NCT05398185) on 5/31/2022.

## Background

Thirty nine million people are currently living with HIV across the globe [1]. While there is no cure for HIV, antiretroviral therapy (ART) can be taken as a daily oral medication, or through longer-acting injections to suppress HIV replication to undetectable levels [2]. People with HIV (PWH) who adhere to their prescribed ART regimen can live a long, healthy life [2]. Despite the efficacy of ART, PWH must take their medication as prescribed to properly manage their HIV.

Although HIV infection rates have decreased globally throughout the past two decades, prevalence rates remain high in certain geographic hotspots [3]. Two settings, New York City (NYC) and La Romana, Dominican Republic (DR), are high priority areas in the US [4] and the Caribbean [5]. The DR has one of the highest rates of HIV infections in the Caribbean with approximately 79,000 PWH [6, 7]. Haiti, with whom DR shares territory on the island of Hispaniola, reports the highest rate in the region [8]. Despite increased accIn the proposed study , we are leveraging a mHealth tool (Wiseess to ART in recent years in the DR, only about 63% of people who have been diagnosed with HIV are taking ART and only 52% have achieved viral suppression (48% virally unsuppressed) [6]. These percentages are lower than in NYC where 84% of PWH have been prescribed ART and 79% are virally suppressed (21% virally unsuppressed) [9]. Regardless, additional interventions are needed to help PWH manage this disease and overcome barriers to achieving viral suppression in both locations. The most recent (2021) NYC HIV/AIDS surveillance report found that of the current 129,660 PWH in NYC, 33.4% self-reported their ethnicity as Latino/Hispanic [9]. This data shows a disproportionate rate of infections among the Latino population as only 28.9% of the total NYC population identifies as Latino [10].

PWH face several barriers to achieving and maintaining medication adherence such as substance use, depression, daily life events, missing refills, side effects, and cost of transportation/medications among others [11]. Mobile health (mHealth) interventions present an equitable solution to receiving tailored care for managing a chronic disease [12, 13]. Although the use of mHealth interventions in DR is still in the early stages of implementation [14], studies have found that 87.6% of people living in the DR own a cellphone [15] with 84% of people of the overall population of the Caribbean owning a smartphone [16], suggesting a high rate of smartphone users in the DR. Meanwhile, a recent study demonstrated that 85% of the United States (US) population owns a smartphone [17], with similar rates estimated in NYC [18]. High smartphone penetration allows for widespread use of mHealth interventions across diseases and populations.

**Fig. 1:**
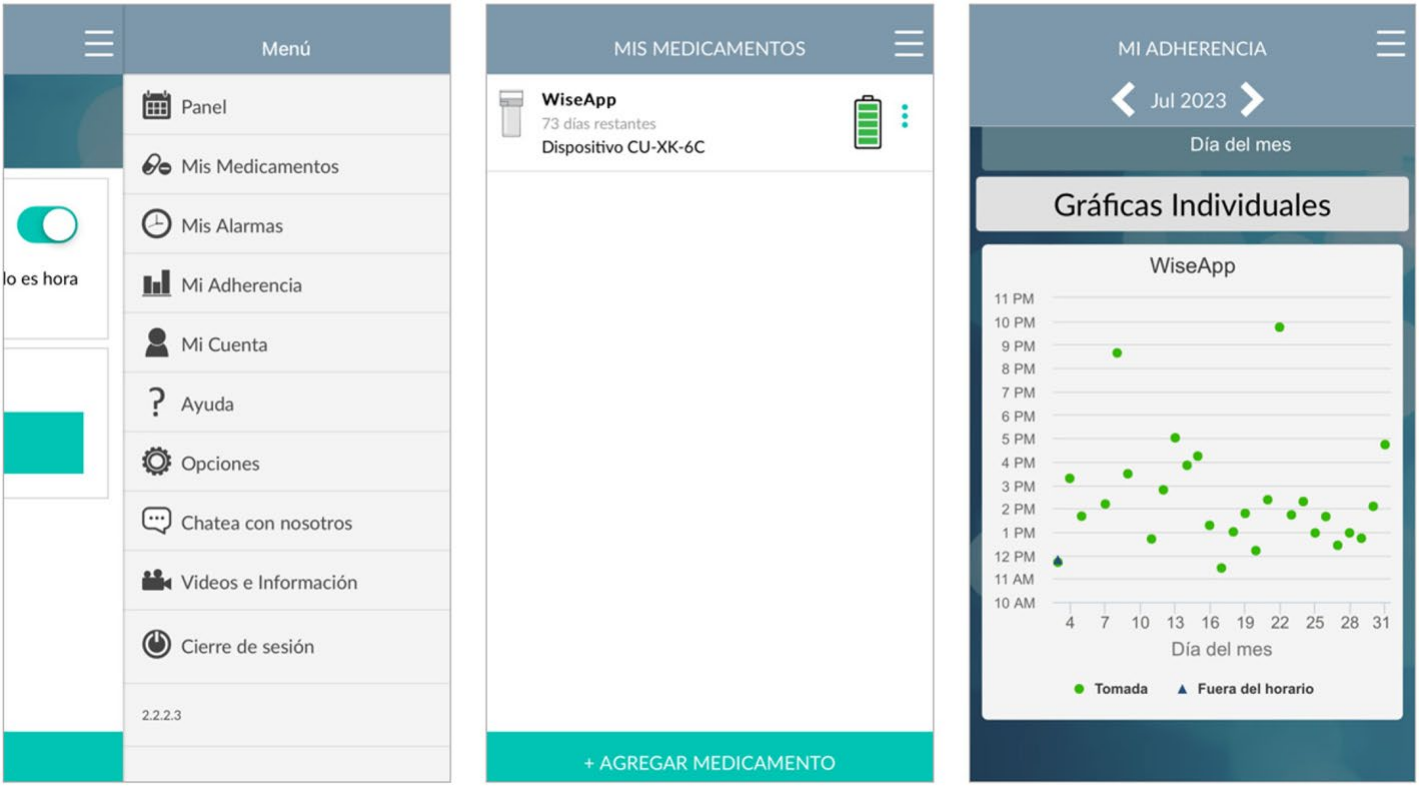
The WiseApp

In the proposed study, we are leveraging a mHealth tool (WiseApp) [19, 20] to test the effectiveness of a smart pill bottle, called the CleverCap, which is an electronic pill bottle that connects via Bluetooth to a medication adherence app (Fig. 1). Through formative work with expert panels, cognitive interviews and usability testing [21, 22], we translated the WiseApp into Spanish and tested it among end-users to refine content to meet the needs of Spanish-speaking Latino/Hispanic PWH in NYC and the DR. Similar studies have been conducted by the study team in NYC [23, 24], however, recruitment did not specifically target Spanish-speakers, and to date, few mHealth interventions have been tested among Spanish-speakers [25, 26]. The objective of this paper is to detail the procedures used for a randomized controlled trial (RCT) which began recruitment in March of 2023.

## Methods

### Study design

This study is a 12-month RCT among Spanish-speaking PWH living in NYC and the DR. Participants are randomized to either receive a CleverCap pill bottle that is linked to the WiseApp (Arm 1), or standard health services offered at each site and a brief adherence educational session (Arm 2). Regardless of randomization, all participants schedule baseline, 3-, 6-, and 12-month visits. Each visit includes taking blood samples (HIV-1 viral load and CD4 counts) and completing an online survey in Spanish. Participants in both arms receive referrals to mental health counseling, drug/alcohol treatment, and/or other HIV services as necessary.

### Recruitment and eligibility

Participants are recruited primarily through direct outreach at the Comprehensive Health Program Adult Services Clinic in the New York-Presbyterian (NYP) Hospital in Washington Heights for the NYC site and at the Clínica de Familia La Romana in La Romana for the DR site. Additional targeted recruitment in NYC occurs through snowball sampling [27], online advertisements, and the distribution of flyers at HIV clinics and community-based organizations with services for PWH and the LGBTQ community. All verbal and written communication during the recruitment process is in Spanish. Those recruited through targeted recruitment are directed to an online web survey via Research Electronic Data Capture (REDCap) for eligibility screening. Eligibility is confirmed through screening in-person or over the phone. In order to be eligible, participants must: (1) speak, read, and write in Spanish; (2) be 18 years or older; (3) be willing to participate in any assigned arm; (4) have an HIV diagnosis of ≥ 6 months ago; (5) have an HIV-1 RNA level > 50 copies/mL; (6) have a viral load > 50; (7) own a smartphone; and (8) provide informed consent for study participation and access to medical records. Participants are not eligible if they: (1) reside in a nursing home, prison, and/or are receiving in-patient psychiatric care at the time of enrollment; (2) have a terminal illness with life expectancy less than six months; (3) plan to move out of the area (NYC or DR) in the next 12 months; (4) have a cognitive state minimum score of 24 measured by the Mini-Mental State Examination (MMSE) [28] to ensure participants are oriented to the time and place; and/or (5) have another family/household member who is already enrolled in the study due to study contamination [29].

### Ethics

All study procedures were approved by the Columbia University Institutional Review Board (IRB) and the Consejo Nacional de Bioética en Salud (CONABIOS), the ethical review committee in the DR, prior to recruitment and enrollment of study participants. Participants review the Informed Consent Form with a study team member and sign consent through REDCap before starting any baseline procedures.

### Sample size calculation

A power analysis was calculated to ensure that the sample size detects at least a medium effect size of the difference in adherence to ART at 3, 6, and 12 months between study arms. We estimated statistical power based on a previous mHealth study where results showed that adherence to ART was higher in the intervention compared to the control arm (0.565 vs. 0.460, that is in the small effect size range) and a Pearson correlation coefficient of adherence of 0.6 between consecutive study times [30]. Using two-sided tests for generalized linear mixed model, we have at least 80% power to test for such differences by site based on these assumptions: (1) target enrollment is 248 participants across sites randomized to each study arm; (2) outcomes are measured at each follow-up visit; and (3) an overestimate of 10% attrition at 3 months and 20% at 6 and 12 months. The sample size at each site does not have enough statistical power to detect a small effect size of difference between the two arms at each follow-up time, but it is enough to test for a medium effect size of difference or greater between the two arms at 3, 6, and 12 months, separately, or to test for a small effect size of difference during entire follow-up period with > 80% statistical power.

### Randomization

Informed consent is obtained at the baseline visit and study participants are randomized to study arms in a 1:1 ratio of 1 - Intervention (arm 1: WiseApp and CleverCap) and 1 - Control (arm 2: standard of care), stratified by site (NYC and DR). Participants are randomized using computer-generated random numbers at baseline and are assigned to one of two trial arms using sequentially numbered, opaque, sealed envelopes containing the intervention assignment, which the staff member opens at the moment of randomization [31].

### Description of the intervention: WiseApp

The WiseApp is an mHealth app [32–34] culturally adapted for Latinos and translated to Spanish through a rigorous iterative community participatory feedback process with the goal of helping PWH adhere to their ART medication regimen. The app is connected to the CleverCap, a smart pill bottle that automatically senses when the bottle is opened and closed, which then records that the user took their medication for the day on the graph within their app account. Participants add their ART to the CleverCap pill bottle at their baseline appointment. Within the app, there are medication reminders and tracking features to set a medication dosing schedule and view adherence statistics. Study participants can also communicate with study staff through the chat feature. The study team is able to view users’ medication adherence statistics through an online portal. More specifically, the study team can view if the participant took their medication on time, off schedule, or if they did not take it. Medication adherence data collected through use of the CleverCap is analyzed upon completion of the study.

### Study assessments

All participants are enrolled on-site at NYP in NYC or Clínica de Familia La Romana, in the DR so that they can complete a blood draw to obtain HIV-1 viral load and CD4 count results at each study timepoint (baseline, 3-, 6-, and 12-months). Participants also complete a survey at all 4 visits. All study materials and visits are completed in Spanish. Surveys are administered through REDCap and are used to collect data on demographics, quality of life, HIV symptoms [35], engagement in HIV care [36], HIV-related stigma [37], alcohol and substance use [38], and depression and anxiety [39–42]. ART adherence is measured through self-report [43] and use of the WiseApp and CleverCap bottle. The differences in study arms are presented in Table 1.

**Table 1:** Intervention v. control procedures and measures

	Baseline	3-Month Follow-Up	6-Month Follow-Up	12-Month Follow-Up
Intervention				
Survey and measures	X	X	X	X
WiseApp & CleverCap		X	X	X
Primary Outcome: ART adherence (SRSI)^a^ [43]	X	X	X	X
Secondary Outcomes: Viral Load/CD4 count through blood draw	X	X	X	X
**Control**				
Survey and measures	X	X	X	X
Standard of care		X	X	X
Primary Outcome: ART adherence (SRSI) [43]		X	X	X
Secondary Outcomes: Viral Load/CD4 count through blood draw	X	X	X	X

^a^Self-report scale item

#### Primary and secondary outcomes

**Fig. 2:**
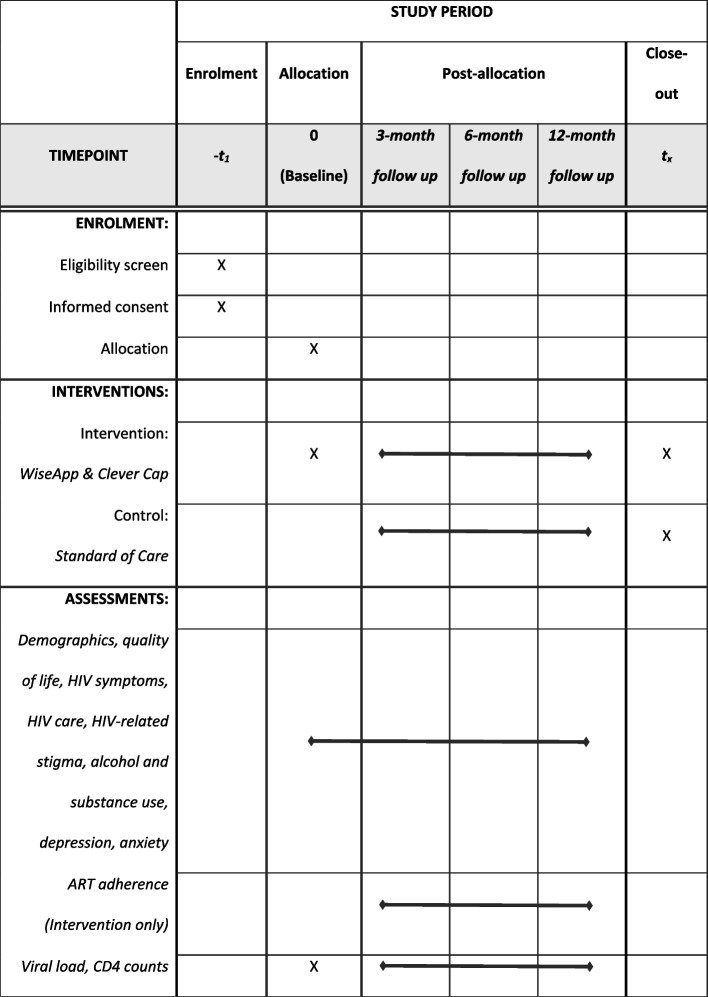
SPIRIT flow diagram for the schedule of enrollment, interventions, and assessments

 The primary outcome is improvement in ART adherence at 3, 6, and 12 months for those randomized to the WiseApp and CleverCap (intervention arm) as compared to the control arm. Measures for ART adherence include: (1) ART adherence (SRSI) [43]; (2) the CleverCap adherence index, which is calculated as the number of unique days that CleverCap indicates either was “Taken” or “Improperly Closed” divided by the number of days during the assessment period; and (3) viral load. The secondary outcome for participants randomized to the intervention arm is (a) improved health-related quality of life; (b) decreased symptom burden; (c) improved engagement with healthcare providers; (d) decreased HIV-related stigma as compared to the control arm; and (e) changes in viral load and CD4 counts using bloods samples taken during study visits. The Standard Protocol Items: Recommendations for Interventional Trials (SPIRIT) flow diagram for the schedule of enrollment, interventions, and assessments of the study are presented in Fig. 2.

### Statistical analysis

Study participants from NYC and DR may be different; therefore, the analyses will be conducted separately for each site as well as for the total data from both sites. Intervention and control arms will be described with respect to baseline characteristics (e.g., means, standard deviations, ranges, and proportions). We will check for outliers, examine patterns of missing data, and conduct evaluation of deviations from normality for all outcomes to determine the need for transformations of variables or special analytic techniques. All multivariate analyses will be preceded by standard descriptive bivariate analyses to describe key variables and relationships among them. These analyses will include means, frequency tables, histograms, and examination of distributions. Following the guidelines issued by the European Medicines Agency [44], we will not adjust for baseline differences in demographic characteristics when comparing the main outcomes between the intervention and control groups. Analyses will be based on linear or generalized linear mixed models. Inferences from mixed models are valid under the missing at random (MAR) assumption (i.e., missingness can be fully accountable by variables where there is complete information) with full-information maximum likelihood (FIML) estimation [45]. These analyses will be complemented with assessment of how sensitive the inferences are to the MAR assumptions. Sensitivity analysis will be performed based on selection models for dropout [46–48]. All analyses will use the Intention-to-Treat principle [49], which requires subjects’ data to be analyzed as randomized regardless of whether or not they used the WiseApp. We will assess for any significant differences in clinical factors (e.g., years since diagnosis, co-morbid conditions), since there is evidence that these clinical factors are related to our primary outcome [50–52]. All statistical tests will be two-sided tests with the level of significance at 0.05.

## Discussion

Many mHealth interventions for medication adherence among PWH have been tested in recent years within the US [53–65] and abroad [66–77]. Past studies have identified usability, acceptability and feasibility of mHealth designed for PWH [53, 55, 60, 61, 67, 69, 73, 74] and some have demonstrated efficacy in improving adherence to ART in the short-term [32, 53, 55, 60, 66, 70, 73, 74]. Among mHealth interventions for PWH in the US, limited studies have recruited or plan to recruit Latino populations specifically [32, 53–55, 57, 58, 60, 61, 63, 64]. An additional limitation to these studies is that of the study samples that included Latino participants, few offered the intervention to those whose first language is Spanish (or, in other words, eligibility criteria includes only English-speakers) [32, 54, 63]. As for the Dominican Republic, there have been very few published health interventions for PWH in recent years [78–80] and more research is needed to identify acceptability of mHealth in low- and middle-income countries [81].

It is estimated that the DR only provides treatment to about 52% of PWH [82], meaning that alternative solutions are needed to ensure that PWH receive continued care to manage their disease. Some factors that affect HIV infection in the DR include poverty, lack of access to healthcare and prevention services, literacy, lack of information on sexuality and HIV/AIDS, and cultural barriers to preventive measures for HIV [7]. mHealth allows for PWH to receive a healthcare intervention on a regular basis when they are unable to see a provider. High smartphone penetration in the DR allows for PWH to engage in mHealth interventions which are well-suited to their needs.

While smartphones are widely used in the US and DR, technology literacy may differ among users, for example, among different age groups. The WiseApp is a simple mHealth application that is to be used primarily to communicate with study staff or view adherence statistics with the purpose of helping PWH take their ART as prescribed. It was created and refined to be a highly usable app for all Spanish-speaking PWH, applying a “Broadcast Spanish” translation, thus avoiding use of colloquial phrases. With the added technology of the CleverCap smart pill bottle, which functions much like a regular pill bottle, participants can easily respond to reminders to take their medication. The WiseApp in Spanish has the potential to reach populations that have been excluded from past interventional studies as well as address disproportionate health outcomes among Latino identifying people.

The WiseApp in Spanish is intended to help PWH better manage their chronic disease by encouraging patients to adhere to a medication dosing schedule. Because of the negative health outcomes which may result from remaining virally unsuppressed, it is crucial that interventions address the specific needs of the target population (Spanish-speaking PWH). Disproportionate rates of HIV infection among Latinos in NYC suggest an existing inequitable approach in reaching and treating this population. Due to a lack of mHealth studies with Latino populations, and apps tailored to Spanish-speakers, the WiseApp study will not only demonstrate the effectiveness of this particular mHealth app, but will also contribute to the mHealth research community as a whole.

## Data Availability

The datasets generated and/or analyzed during the current study contain sensitive personally identifiable information, including participant’s name and HIV status, and are not publicly available. Study information would be available from the corresponding author upon reasonable request.

## Abbreviations

PWH: People with HIV
ART: Antiretroviral therapy
NYC: New York City
DR: Dominican Republic
RCT: Randomized controlled trial
NYP: New York-Presbyterian
MMSE: Mini-Mental State Examination
SRSI: Self-report scale item
MAR: Missing at random
FIML: Full-information maximum likelihood
